# Acute Aortic Regurgitation After Transcatheter Aortic Valve Implantation Procedure

**DOI:** 10.7759/cureus.50345

**Published:** 2023-12-11

**Authors:** Bothayna Amien, Clare Appleby, Joe Mills, Kully Sandhu, Deborah Harrington

**Affiliations:** 1 Department of Cardiothoracic Surgery, Liverpool Heart and Chest Hospital, Liverpool, GBR; 2 Department of Cardiology, Liverpool Heart and Chest Hospital, Liverpool, GBR

**Keywords:** transcatheter aortic valve implantation (tavi), cardiac tamponade, pericardial effusion, aortic valve replacement (avr), paravalvular leak, aortic regurgitation

## Abstract

This study reports the case of a 75-year-old woman who developed aortic regurgitation (AR) a few hours after transcatheter aortic valve implantation (TAVI). The patient underwent the TAVI procedure for aortic stenosis and became hypotensive in recovery. A transthoracic echo revealed cardiac tamponade and around 1500 ml of blood was drained over several hours. Further advice was sought from the surgical team, and a transoesophageal echo revealed significant AR, which was confirmed by a transthoracic echo performed the next day. The patient underwent an emergency surgical aortic valve replacement.

This case study demonstrates one of the complications of the TAVI procedure, acute AR, which was diagnosed a few hours after the procedure.

## Introduction

The use of transcatheter aortic valve implantation (TAVI) in treating severe aortic stenosis has grown rapidly. TAVI offers many surgically unfit patients hope for a better outcome than with medical treatment alone [1]. With more success and increased technical experience, TAVI also has an increasing trend towards implantation in lower-risk patients. Despite favourable overall success rates, TAVI can still result in significant complications [1].

This report details the case of a surgically fit 75-year-old woman who underwent TAVI and required surgical valve replacement during the same admission due to significant aortic regurgitation (AR) following the procedure. In this case report, we will discuss the AR post-TAVI in more detail and the proposed mechanisms that could lead to this complication.

This article was previously presented as a meeting abstract at the 2023 ESSR European Society of Surgical Research Meeting on June 30, 2023.

## Case presentation

This study concerns a 75-year-old female, otherwise fit, with a history of worsening dyspnoea on exertion. Investigations revealed severe aortic stenosis with a mean gradient of 46 mmHg and an indexed aortic valve area of 0.3 cm2/m2 on a transthoracic echocardiogram. An exercise tolerance test was positive, and coronary angiography revealed non-significant coronary disease. The patient was presented with the option of open surgical aortic valve replacement but declined this in favour of a TAVI procedure due to the anticipated shorter recovery time.

An Acurate neo2 valve measuring 23 mm (Boston Scientific Corporation, Massachusetts, USA) was inserted using the transfemoral technique. The valve looked to be well-positioned angiographically, with only slight AR and favourable haemodynamics, in accordance with conventional operating standards. The patient developed hypotension two to three hours after being transferred to recovery. A transthoracic echo showed cardiac tamponade and a pericardial effusion. After a pericardial drain was inserted, about 1500 mL of blood was drained over several hours; hence, surgical guidance was sought. A CT scan showed a contrast leak and haemostasis from the left ventricle's lateral wall but no signs of aortic damage. The patient was transferred to the theatre for exploration, and a transoesophageal echo revealed significant AR (Video 1). Following a multidisciplinary team discussion, a surgical subxiphoid pericardial drain was inserted, and the patient was stabilised and awakened. A further multidisciplinary discussion occurred the following day, and another transthoracic echo confirmed severe transvalvular AR.

**Video 1:** TOE showing severe AR post-TAVI insertion TOE: transoesophageal echo, TAVI: transcatheter aortic valve implantation, AR: aortic regurgitation

The patient underwent an emergency median sternotomy, a TAVI explant, and an open aortic valve replacement using a 21 mm Carpentier-Edwards Perimount Magna Ease aortic valve (Edwards Lifesciences, Irvine, CA). The lateral wall of the left ventricle was covered by a small haemostasis, but no active bleeding was present; thus, the haemostasis was left undisturbed. The operation was performed without incident, but unfortunately, the patient developed a dense left-sided weakness on waking. An MRI scan of the brain revealed bilateral watershed infarcts. The patient made a slow recovery and was transferred for stroke rehabilitation.

## Discussion

The patient in this study had several events; pericardial effusion post-TAVI and a contrast leak from the lateral wall of the left ventricle were revealed on a CT scan. The leak was probably a left ventricle wire tip perforation that healed conservatively, and as of exploration, no bleeding was identified from the left ventricle. The transoesophageal echo revealed significant AR, which necessitated surgical intervention. After the successful replacement of the valve, the patient developed left-sided weakness on waking and was unfortunately diagnosed with stroke following an MRI scan.

AR is a significant complication of TAVI, which can hugely impact patients’ recovery and affect mortality in the long term. Moderate or severe AR after TAVI is associated with an increased risk of mortality [2,3]. Therefore, it is crucial to pay close attention to these risks and thoroughly assess patients, particularly those with low surgical risk.

The leakage can be in the form of total AR or paravalvular regurgitation [3]. AR is usually attributed to either failure of the prosthesis [4,5] or underexpansion of the valve, usually after implanting a large valve into a small annulus [4,6]. Paravalvular regurgitation is caused by the implantation of a small prosthesis into a large annulus and either a too-high or too-low valve implantation [4,7].

The choice of valve can also affect the development of leakage post-TAVI [3]. The most commonly used valve groups are self-expandable valves (SEV) and balloon-expandable valves (BEV). Kooistra et al., comparing balloon-expandable Sapien 3 Ultra vs. a self-expandable CoreValve, showed that AR was lower in the BEV group than in the SEV group, with a regurgitant fraction of 1.1% (0-8.0) vs. 8.7% (3.0-14.8). Very little data exists to compare the latest generations of both groups. Pellegrini et al. compared the latest generation (SEV) Acurate neo2 with (BEV) Sapien 3 Ultra; both showed great overall outcomes. However, paravalvular regurgitation was significantly lower with Sapien 3 Ultra [8].

After an external review of the patient, it was concluded that the likeliest cause of the problem was the initial implantation being high (Videos 2-3), possibly with subsequent further microembolisation. Imaging reviews raised the possibility of leaflet deformity, but no mechanical defect was identified on the explant, which could support suboptimal expansion in a high position.

**Video 2:** TOE showing TAVI valve TOE: transoesophageal echo, TAVI: transcatheter aortic valve implantation The video below is taken from the pre-operative TOE showing the TAVI valve

**Video 3:** TOE showing TAVI valve migrated TOE: transoesophageal echo, TAVI: transcatheter aortic valve implantation The video below is taken from the pre-operative TOE showing the TAVI valve in a slightly higher position when compared to Video 2

## Conclusions

AR is a significant complication post-TAVI that can severely impact mortality; therefore, careful assessment of the patient is required, and consideration of the type of valve used is also essential.

The patient in this study had a small left ventricular outflow tract, and suboptimal expansion of the TAVI valve in a high position likely contributed to the subsequent AR, highlighting the importance of a BEV versus an Acurate neo2 valve.

This case stresses the importance of multidisciplinary heart team assessment, particularly where there is clinical equipoise or low surgical risk for which the gold-standard operation remains open aortic valve replacement.
